# Association of common genetic variants with chronic axonal polyneuropathy in the general population: a genome-wide association study

**DOI:** 10.3389/fneur.2024.1422824

**Published:** 2024-07-03

**Authors:** Noor E. Taams, Maria J. Knol, Rens Hanewinckel, Judith Drenthen, Mary M. Reilly, Pieter A. van Doorn, Hieab H. H. Adams, M. Arfan Ikram

**Affiliations:** ^1^Department of Epidemiology, Erasmus University Medical Center, Rotterdam, Netherlands; ^2^Department of Neurology, Erasmus University Medical Center, Rotterdam, Netherlands; ^3^Department of Neurology, Canisius Wilhelmina Hospital, Nijmegen, Netherlands; ^4^Department of Clinical Neurophysiology, Erasmus University Medical Center, Rotterdam, Netherlands; ^5^Centre for Neuromuscular Diseases, Department of Neuromuscular Diseases, UCL Queen Square Institute of Neurology and National Hospital for Neurology and Neurosurgery, London, United Kingdom; ^6^Department of Human Genetics, Radboud UMC, Nijmegen, Netherlands; ^7^Latin American Brain Health (BrainLat), Universidad Adolfo Ibáñez, Santiago, Chile

**Keywords:** GWAS, genetics, common variants, chronic axonal polyneuropathy, neuropathy

## Abstract

**Introduction:**

Disease susceptibility of chronic axonal polyneuropathy is not fully explained by clinical risk factors. Therefore, we determined the contribution of common genetic variants in chronic axonal polyneuropathy in the general population.

**Methods:**

This study was performed in two population-based studies. Polyneuropathy diagnosis was based on screening in the Rotterdam Study and on ICD-10 codes in the UK Biobank. We determined the heritability of the sural nerve amplitude and performed genome-wide association studies of chronic axonal polyneuropathy and sural sensory nerve amplitude. Furthermore, we zoomed in on variants in and surrounding 100 autosomal genes known to cause polyneuropathy based on literature and expert knowledge (candidate genes), and we performed a gene-based analysis. Analyses were adjusted for age, sex and population stratification.

**Results:**

Chronic axonal polyneuropathy was present in 2,357 of the 458,567 participants and 54.3% of the total population was female. Heritability of sural nerve amplitude was 0.49 (*p* = 0.067) (*N* = 1,153). No variants (*p* < 5.0×10^−8^) or genes (*p* < 2.7×10^−6^) reached genome-wide significance for its association with polyneuropathy. Focusing on variants in and surrounding the candidate genes in the GWAS (*p* < 3.9×10^−6^) and on these genes in the gene-based analysis (*p* < 5.0×10^−4^) neither yielded significant results.

**Discussion:**

We did not find common variants associated with chronic axonal polyneuropathy in the general population. Larger studies are needed to determine if genetic susceptibility based on both common and rare genetic variants affect the risk for chronic axonal polyneuropathy in the general population.

## Introduction

Chronic axonal polyneuropathy is a common disabling disease with a prevalence of 4.0–7.0% that increases with age (1–3). The usual presentation of this complex disease is a slowly progressive, symmetric polyneuropathy resulting in sensory disturbances and sometimes loss of strength. The etiologic mechanisms of chronic axonal polyneuropathy are only partially understood, and numerous environmental risk factors have been identified of which diabetes, alcohol abuse and vitamin deficiencies are the most common (4, 5). Besides environmental factors, genetic factors may also contribute to disease risk.

The importance of genetic variation is known for inherited neuropathies, e.g., Charcot–Marie-Tooth (CMT) disease, in which mostly a rare, monogenic mutation with a high penetrance leads to the development of the disease (6). Furthermore, common and rare genetic variants have been detected in chronic idiopathic axonal polyneuropathy and in chemotherapy-induced and diabetic polyneuropathy (7–11). Common genetic variants can lead to susceptibility for complex polygenic diseases. Those variants are relatively frequent in the general population with a low penetrance (12), and may therefore only be detectable at population level. The high prevalence of non-hereditary chronic axonal polyneuropathy in the general population suggests that genetic components may in part be due to a large number of common genetic variants each with relatively small effects (13).

In this study, we performed four consecutive analyses: first, we determined the heritability of the sural sensory nerve action potential (SNAP) amplitude; second, we performed a genome-wide association study (GWAS) on chronic axonal polyneuropathy and sural SNAP amplitude; third, we zoomed in on genes known to cause CMT (candidate genes) within the GWAS analysis and fourth, we conducted gene-based analyses for the same outcomes.

## Materials and methods

### Study population

The population consisted of participants from two population-based studies, namely the Rotterdam Study (14) and the UK Biobank (http://www.ukbiobank.ac.uk). Combining these two studies increases the number of participants and therefore the power needed to perform a GWAS. Participants in the Rotterdam study were recruited based on their age (≥40 years old) and residence in a district of Rotterdam, the Netherlands (14). Participants of the UK Biobank were recruited between 2006 and 2010, aged 40 to 69 years old and living in the United Kingdom.

Participants from both studies were eligible if their polyneuropathy status, irrespective of the cause or risk factors, and genetic data were available (N_Rotterdam Study_ = 1,567, N_UK Biobank_ = 457,218). Participants with known hereditary polyneuropathy (N_Rotterdam Study_ = 2; N_UK Biobank_ = 216) were excluded, resulting in a study population of 1,565 participants from the Rotterdam Study and 457,002 from the UK Biobank. Sural sensory nerve action potential (SNAP) amplitude was only available for 1,153 participants of the Rotterdam Study (N_missing_ = 412). Both population-based studies were approved by institutional review boards or equivalent organizations, and all participants provided written informed consent.

### Genotyping and quality control

Genotyping was performed with commercially available arrays for common genetic variants. In the Rotterdam Study the Illumina 550 K, 550 K duo or 610 quad array (14) were used. Samples were removed if the call rate was below 97.5%, as well as gender mismatches, excess autosomal heterozygosity, family relations, ethnic outliers, duplicates, variants with a call rate lower than 95%, failing missingness test, Hardy–Weinberg equilibrium *p* < 10^−6^ and allele frequencies below 0.01. We also excluded genetic variants with low imputation qualities (*r*^2^ < 0.3). Additionally, genetic variants were removed if the following filter was equal or below five: imputation quality (INFO or r^2^) * minor allele frequency * number of cases (for polyneuropathy) or total number of individuals (for sural SNAP). Genotypes were imputed to the Haplotype Reference Consortium v1.1.

In the UK Biobank, genotyping was performed using the Affymetrix UK BiLEVE array (*N* ~ 50,000) and the Affmetrix UK Biobank Axiom Array (*N* ~ 450,000). The quality control procedure has been described in more detail elsewhere (15). In short, participants were genotyped in 106 batches, and each QC procedure was performed for every batch separately. To account for population structure, sample-based and marker-based QC have been adapted based on principal component analysis. Genetic markers were tested for batch and plate effects, departure from Hardy Weinberg equilibrium, sex and array effects and discordance across control replicates. If a marker failed one of the tests, it was set to missing in that specific batch. Based on the sample QC, we removed samples identified as outliers in heterozygosity and missing rates, and samples identified as putatively carrying sex chromosome configurations other than XX or XY. Genotypes were imputed to the Haplotype Reference Panel (HRC) version 1.1, and additionally to the UK10K and 1,000 Genomes reference panels (15).

### Polyneuropathy diagnosis

In the Rotterdam Study, polyneuropathy screening consisted of three components including a symptoms questionnaire, neurological examination of the legs and nerve conduction study (NCS) of the sural nerves (1, 2). The questionnaire consisted of symptoms that are related with polyneuropathy, that occur bilaterally for at least 3 months and answer could be never, sometimes or (almost) continuously, as described in detail elsewhere (2, 16). Neurological examination consisted of a bilateral examination of the legs including tendon reflexes, dorsal flexion of the feet and several sensory tests (vibration and superficial pain sensation). NCS were performed with a Nicolet™ Viking Quest (Natus Medical Incorporated, San Carlos, California, United States). The sural nerve was antidromically measured with surface electrodes, SNAP amplitudes were measured from baseline to peak, and sural SNAP amplitude <4.0 μV was considered abnormal (2, 17). This cut-off value, irrespective of age, may have led to an underdiagnosis in younger participants and overdiagnosis in elderly participants. For interpretation, the highest of both sural SNAP amplitudes was used for analyses. All participants were categorized as ‘no’, ‘possible’, ‘probable’ or ‘definite’ chronic axonal polyneuropathy, irrespective of their cause. Categorization was determined by discussion in an expert panel (authors PD, JD, NT and RH) and based on the abnormalities of our screening. Abnormalities in components of the screening were symptoms and neurological signs corresponding with polyneuropathy and sural SNAP amplitudes below <4.0 μV, as described in detail elsewhere (2). Participants were excluded if ≥2 of the components were missing. Additionally, their medical records were reviewed for diagnosis of polyneuropathy by a neurologist, as this was considered superior to our screening (2). In the current study, ‘definite’ and ‘probable’ polyneuropathy were combined into ‘polyneuropathy’ because of their clinical similarities and ‘possible’ and ‘no’ polyneuropathy were combined into ‘controls’.

In the UK Biobank, diagnosis of polyneuropathy was based on ICD-10 codes present in their medical records. Participants with ICD-10 codes G60.3 (idiopathic progressive), G62 (other polyneuropathies, e.g., drug-induced and alcoholic) and G63 (polyneuropathy in diseases classified elsewhere, e.g., diabetes and nutritional deficiency) were included. Individuals known with hereditary and inflammatory neuropathies were excluded from the analyses (ICD-10 codes G60 and G61, respectively).

To increase statistical power, we created an extra outcome variable which also included the presence of self-reported peripheral neuropathies from the UK Biobank. This was reported using questionnaires.

## Analysis

### Heritability

Using the Genome-wide Complex Trait Analysis (GCTA) software (18, 19) we estimated the heritability of the sural SNAP amplitude, a direct correlate of chronic axonal polyneuropathy, in the Rotterdam Study (N = 1,153). Using this GCTA-GREML method we compare the genetic similarity between phenotypes to estimate the variance explained by genetics. Due to the limited power, we were not able to assess the heritability of the dichotomous polyneuropathy variable and similarly, we were not able to estimate the SNP-based heritability using GWAS summary statistics.

### Genome-wide association study

GWAS was performed using logistic regression under an additive model for polyneuropathy in 458,567 participants, and using linear regression under an additive model for the sural SNAP amplitude in 1,153 participants. In the Rotterdam Study, these analyses were performed with the RVTESTS software (20) and in the UK Biobank with the SAIGE software (21). Adjustments were made for age, sex and principal components, and in the UK Biobank additionally for genotyping array. Hereafter, genetic variants were removed if the following filter was equal or below five: imputation quality (INFO or *r*^2^) * minor allele frequency * number of cases (for polyneuropathy) or total number of individuals (for sural SNAP). Meta-analyses of these results were performed in METAL (22) using an inverse-variance weighted fixed effects model with a standard error analysis scheme.

### Focus on (candidate) genes

We determined candidate genes based on literature and expert knowledge of hereditary neuropathies. In collaboration with the Centre for Neuromuscular Diseases, UCL Queen Square Institute of Neurology and National Hospital for Neurology and Neurosurgery, London we defined a set of 110 genes that are associated with hereditary polyneuropathy (Supplementary Table S1). Of those, ten genes were excluded from the analysis as they were located on the X-chromosome (i.e., *AR, ATP7A, GJB1, AIFM1, PDK3, LAS1L, DRP2* and *PRPS1*) or were mitochondrial genes (*MTATP6* and *MTATP8*). This resulted in 100 candidate genes to target in our analyses in 458,567 participants. In the GWAS analysis, we zoomed in on genetic variants located within a distance less than 50 kb outside the 100 selected genes (N_variants_ = 45,573) and we used permutation testing (*N* = 10,000) to calculate the number of independent genetic variants. This resulted in a *p*-value threshold for significance of *p* < 3.9×10^−6^ (0.05/12,801 independent genetic variants).

We additionally performed a gene-based analysis using MAGMA (23), which aggregates the effects of multiple variants within a gene. The GWAS summary statistics was used as the input dataset with the 1,000 Genomes European sample as a reference set and a *p*-value threshold for significance of *p* < 2.7×10^−6^ (0.05 / 18,339 genes). In this analysis too, we zoomed in on the 100 candidate genes for polyneuropathy, using a p-value threshold for significance of *p* < 5×10^−4^ (0.05/100 genes).

Furthermore, we aimed to investigate whether the common genetic variants identified in a previous GWAS for idiopathic form of polyneuropathy also showed an association in our sample. Hence, we performed a look-up for rs7294354 and rs147738081 identified by Winsvold et al. (10).

## Results

### Study population

In total, 458,567 participants were included with an average age of 57.3 ± 8.0 years (*Age_RotterdamStudy_* 73.6 ± 9.1 years; Age*_UKBiobank_* 57.3 ± 8.0 years) and 54.3% was female [*N_RotterdamStudy_* = 837 (53.5%); *N_UKBiobank_* = 248,331 (54.3%)]. Polyneuropathy was present in 2,357 participants (*N_RotterdamStudy_* = 215, *N_UKBiobank_* = 2,142) and median sural SNAP amplitude was 8.0 [interquartile range (IQR) 5.0–11.0] (Table 1).

**Table 1:** Study population characteristics.

	Rotterdam study (*N* = 1,565)	UK Biobank (*N* = 457,002)
**Female**, *n* (%)	837 (53.5)	248,331 (54.3)
**Age**, years	73.6 ± 9.1	57.3 ± 8.0
**Polyneuropathy**, *n* (%)	–	2,142 (0.47)
Definite and probable	215 (13.7)	–
Possible and no	135 (86.3)	–
**Self-reported neuropathy**, *n* (%)	–	497 (0.11)
**SNAP amplitude**, median (IQR) ^&^	8 (5.0–11.0)	–

*N*: number, SNAP: sural sensory nerve action potential, IQR: interquartile range, −: not applicable, &: available in 1153 participants.

### Heritability and genome-wide association study

The heritability estimate for sural SNAP amplitude was 49% (*p = 0.067*).

**Figure 1:**
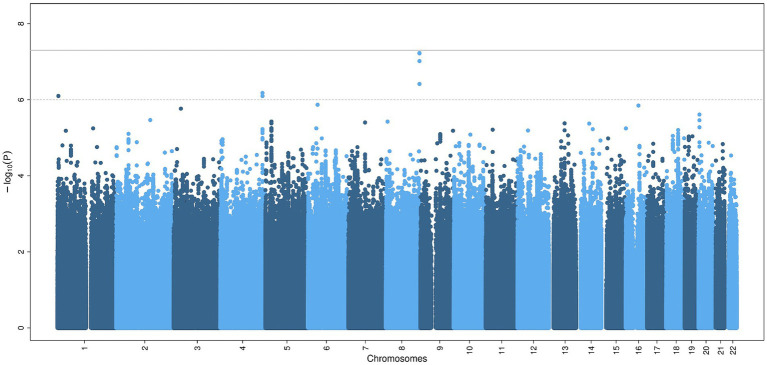
Common genetic variants associated with chronic axonal polyneuropathy. Legend: Manhattan plot of the genome-wide association study for chronic axonal polyneuropathy. The solid line represents the significance threshold for all genetic variants (*p < 5×10^−8^*), the dotted line the significant threshold for genetic variants in or nearby (± 50 kb) candidate genes (*p < 1×10^−6^*).

The GWAS did not show statistically significant variants (*p < 5×10^−8^*) (Figure 1), nor after including self-reported peripheral neuropathies in the UK Biobank (Supplementary Figure S1). Variants with borderline significance (*p < 5×10^−6^*) are listed in Table 2. Three of the most significant common variants were located in the gene AC138647.1 on chromosome 8 (lead variant rs34077186, *p < 5.9×10^−8^*). This is a protein coding gene, associated with chronic pain (24). Other genes listed in Table 2 are associated with body mass index, type 2 diabetes and Parkinson’s disease.

**Table 2:** Association of independent genetic variants suggestively (*p < 5×10^−6^*) associated with chronic axonal polyneuropathy.

SNP	Chr	Nearest gene	A1	A2	Frequency allele 1	Beta	SE	*p*-value
rs34077186	8	*AC138647.1*	CGA	C	0.966	−0.487	0.090	5.9×10^−8^
rs1676205	4	*U1*	A	G	0.815	−0.194	0.039	7.6×10^−7^
rs61768776	1	*RP13-614 K11.1*	A	G	0.070	−0.310	0.063	8.0×10^−7^
6–40,807,644	6	*LOC105375053*	A	AT	0.152	0.216	0.045	1.4×10^−6^
rs71323034	3	*hsa-mir-466*	A	G	0.012	0.707	0.148	1.7×10^−6^
rs147852732	20	*SMOX*	T	C	0.014	0.653	0.139	2.5×10^−6^
rs201447356	2	*ARHGAP15*	T	TA	0.328	0.170	0.037	3.4×10^−6^

Within a genetic locus, the most significant SNP is noted. Abbreviations: SNP: single nucleotide polymorphism, Chr: chromosome, A1: effect allele, A2: other allele, SE: standard error.

### Focus on (candidate) genes

**Figure 2:**
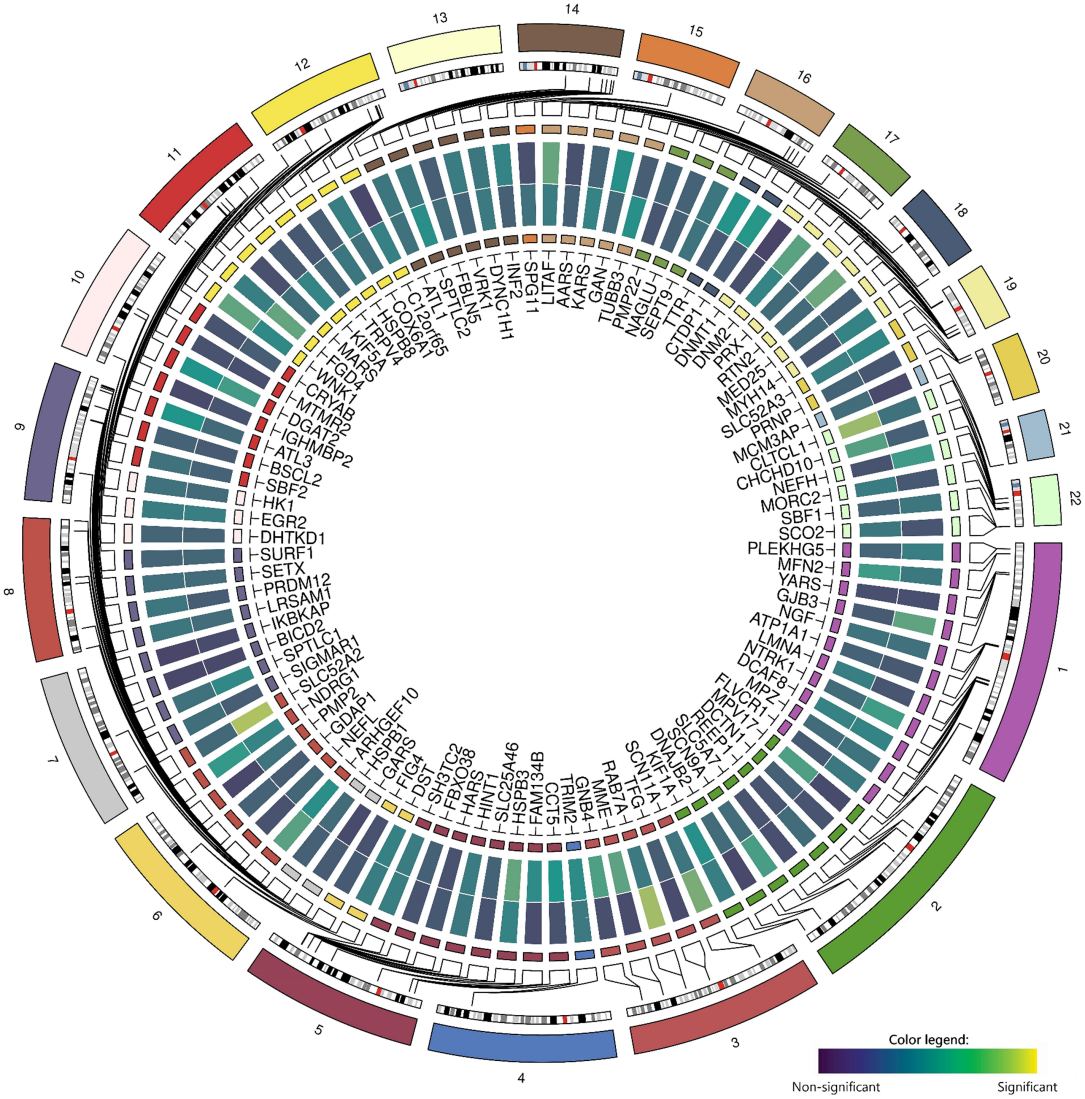
Associations between common genetic variants in the 100 candidate genes with chronic axonal polyneuropathy (*N* = 458,567) and sural sensory nerve action potential study (*N* = 1,153). Legend: Circos-heatmap representing *p*-values of the most significant SNP per candidate gene, colored from blue (not significant) to yellow (significant: *p < 3.9×10^−6^*). The inner heatmap represents the association with sural sensory nerve action potential, the outer heatmap represents the association with chronic axonal polyneuropathy.

Zooming in on genetic variants within and near the 100 candidate genes (± 50 kb) within the GWAS did not yield significant results for chronic axonal polyneuropathy nor for sural SNAP amplitude (Figure 2).

**Figure 3:**
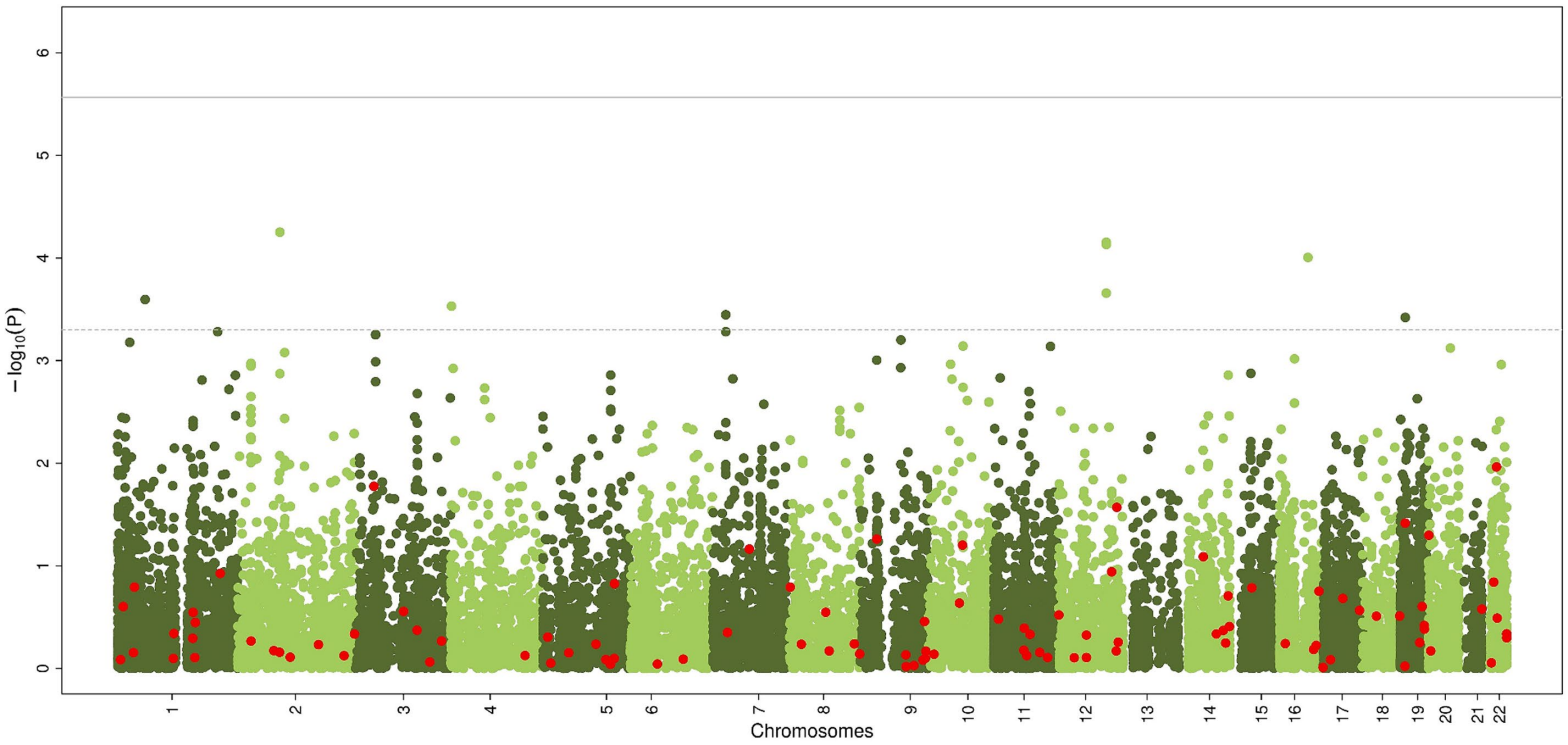
Common genetic variants, aggregated within genes, associated with chronic axonal polyneuropathy using generalized gene-set analysis of GWAS data (MAGMA). Green dots represent all genes, red dots represent the 100 candidate genes. Solid line represents significance threshold for all genes, dotted line for significant threshold of candidate genes.

The gene-based analysis using MAGMA is shown in Figure 3. None of the genes reached statistical significance, neither when zooming in on the candidate genes (most significant gene is *PLGB1*, *p < 5.61×10^−5^*).

The previously identified genetic variants by Winsvold et al. (10) for chronic idiopathic axonal polyneuropathy were associated with polyneuropathy in our sample at a nominal significance level but did not survive the Bonferroni significance threshold (p_rs7294354_ = 0.0213; p_rs147738081_ = 2.27×10^−3^).

## Discussion

In this study, consisting of two population-based studies, we found a moderate to high heritability for the sural SNAP amplitude, albeit not statistically significant (*p = 0.067*). We did not identify common genetic variants for the non-hereditary forms of chronic axonal polyneuropathy in the GWAS, neither using the gene-based analysis nor when zooming in on the candidate genes.

We did not find common variants associated with chronic axonal polyneuropathy and were not able to replicate previously identified susceptible loci for idiopathic polyneuropathy. We first discuss our results in perspective to the literature, and subsequently we elaborate on potential explanations for the null-finding of our study.

Recently, a GWAS (UKB and Norwegian registry data) on chronic idiopathic axonal polyneuropathy identified two susceptible loci in a meta-analysis (10). We did not detect the same loci although both studies used data from the UKB. Therefore, it is important to elaborate on possible explanations for the different findings. One explanation concerns the investigated phenotypes; the previous GWAS (10) included solely patients with chronic idiopathic axonal polyneuropathy, whereas our study included a more heterogeneous population of participants with chronic axonal polyneuropathy irrespective of the cause (excepting hereditary). Another explanation is the difference in diagnosing polyneuropathy. In the previous GWAS polyneuropathy diagnosis was solely based on ICD-10 codes, resulting in the very low prevalences as probably only severe cases are included, which is possibly not representative of cases in the general population. In the Rotterdam Study, part of our analysis, the prevalence of chronic axonal polyneuropathy resembled the population prevalence, that includes both mildly and severely affected persons with a range of different axonal polyneuropathies (1, 25, 26). It is also possible that the genomic loci in the previous GWAS were mainly driven by the Norwegian samples, resulting in a non-significant finding in our study. Furthermore, it is not excluded that the identified loci were false-positive findings, since no replication study was performed to validate the results of that study.

Now, we will elaborate on potential explanations for the null-finding within our study. Firstly, environmental risk factors rather than common genetic factors may predominantly contribute to the development of chronic axonal polyneuropathy. Indeed, previous research found multiple environmental risk factors to be associated with polyneuropathy in a general population, in particular diabetes mellitus, vitamin deficiencies and alcohol abuse (4, 27). In contrast, common genetic variants for chronic axonal polyneuropathy have mostly been detected in specific, regularly homogenous, high-risk group of patients with type 2 diabetes or patients receiving chemotherapy (8, 9, 28) and the yield is higher in familial and hereditary cases than the non-hereditary cases (7, 29). Although the heritability analysis of the sural SNAP amplitude suggests otherwise, a potential explanation for our null finding may be that environmental risk factors are the main driver with a limited role of common genetic factors.

Secondly, our finding can be explained because rare variants either single or multiple, rather than common variants, influence the genetic susceptibility for polyneuropathy. Recently, rare variants in *MME* were detected in both familial and non-familial cases of chronic idiopathic axonal polyneuropathy (7, 28), and repeat expansions of *RFC1* in patients with a complex phenotype (sensory neuropathy, cerebellar ataxia and vestibular disturbance) and also in patients with idiopathic sensory neuropathy although with time this may evolve to the more complex phenotype (30, 31). We did not find significant variants in *MME* or *RFC1*, but this may be explained by the difference in patient populations and our focus on common rather than rare variants (7, 30–32). We performed a GWAS in a population-based sample without hereditary polyneuropathies to focus on common variants (MAF > 1%), instead of whole exome sequencing (WES) that focuses on rare variants within *MME*. Furthermore, we did not use molecular genetic testing to detect nucleotide repeat expansions in *RFC1* (32). Future studies using whole-genome sequencing or whole-exome sequencing may be able to detect rarer genetic variants and/or repeat expansions influencing the risk of chronic axonal polyneuropathy.

Thirdly, methodological constraints should be taken into account. Most important, our GWAS had limited power with only 2,357 cases (0.5%) across the two studies. Yet, we note that studies used for our analyses are the only population-based studies with polyneuropathy and genome-wide genetic data to our knowledge. The prevalence of polyneuropathy in the Rotterdam Study (13.7%) is relatively high and in the UKB (0.47%) low compared to similar populations from population-based studies (4.0–7.0%) (1–3). The higher prevalence in the Rotterdam Study is a consequence of the methodological consideration to increase the number of cases by combining definite (met all three criteria for diagnosis) and probable (typical signs of polyneuropathy) as the group of interest (Table 1). The low prevalence of polyneuropathy in the UKB is probably because the diagnosis was based on ICD-10 codes from hospital and/or GP records, and additionally may also partly be explained by the lower mean age. This may have resulted in an underestimation of the number of cases and a dilution of the effects. We acknowledge that the current number of cases would have allowed us to detect only common variants with a relatively large effect. To increase the number of cases, we performed a sensitivity analysis including the cases with self-reported polyneuropathy. These results did not yield new results, probably as self-reported polyneuropathy is often unreliable (1). In addition, due to the limited power in our study we were not able to estimate the heritability of polyneuropathy. In a larger sample it would however be interesting to assess the SNP-based heritability using GWAS summary statistics. Although we were able to calculate the heritability for the sural SNAP amplitude using GCTA (18), this data was only available in a small sample (*N* = 1,072) and its non-significant estimate should therefore be treated with caution.

Fourthly, ethnic differences may influence the susceptibility for chronic axonal polyneuropathy. Prevalence of chronic axonal polyneuropathy differs between populations and countries with a higher prevalence in European countries than in African and Middle Eastern countries (4). This difference might be explained by underdiagnoses as access to healthcare in the countries with lower prevalence may be limited, by the use of different diagnostic protocols or because of different prevalence of risk factors. However, the differences could also be driven by genetics as is suggested for hereditary polyneuropathies (33).

In conclusion, we did not identify common genetic variants that were associated with chronic axonal polyneuropathy in the general population. Further studies are needed to identify both rare and common variants as the phenotype of non-hereditary chronic axonal polyneuropathy is still not fully explained by the known environmental risk factors. Preferably these larger studies should be conducted in populations that are carefully screened for the presence of chronic axonal polyneuropathy based on the assessment of physical complaints, neurological examination and nerve conduction studies, all with the aim to unravel the impact of environmental risk factors and genetic predisposition in chronic axonal polyneuropathy.

## Data availability statement

Publicly available datasets were analyzed in this study. Data from the Rotterdam Study can be obtained on request directed to the management team (secretariat.epi@erasmusmc.nl), which has a protocol for approving data requests. Data from the UK Biobank (UKB) can be accessed upon application (https://www.ukbiobank.ac.uk/).

## Ethics statement

The studies involving humans were approved by the Medical Ethics Committee of Erasmus MC (registration number MEC 02.1015) and by the Dutch Ministry of Health, Welfare and Sport (Population Screening Act WBO, license number 1071272-159521-PG). The UK Biobank has obtained Research Tissue Bank (RTB) approval from its governing Research Ethics Committee (REC), as recommended by the National Research Ethics Service (NRES). The studies were conducted in accordance with the local legislation and institutional requirements. The participants provided their written informed consent to participate in this study.

## Author contributions

NT: Conceptualization, Data curation, Investigation, Methodology, Resources, Visualization, Writing – original draft, Writing – review & editing. MK: Data curation, Formal analysis, Investigation, Methodology, Resources, Software, Writing – original draft, Writing – review & editing, Visualization. RH: Data curation, Writing – original draft, Writing – review & editing, Resources. JD: Data curation, Resources, Writing – original draft, Writing – review & editing. MR: Writing – original draft, Writing – review & editing, Methodology. PD: Conceptualization, Funding acquisition, Supervision, Writing – original draft, Writing – review & editing, Methodology. HA: Methodology, Supervision, Writing – original draft, Writing – review & editing, Resources. MI: Conceptualization, Funding acquisition, Methodology, Supervision, Writing – original draft, Writing – review & editing.

## Supplementary material

SUPPLEMENTARY FIGURE 1 Association of common genetic variants with chronic axonal polyneuropathy, including self-reported peripheral neuropathies in the UK Biobank. Manhattan plot showing the genome-wide association study for chronic axonal polyneuropathy, including the self-reported peripheral neuropathies in the UK Biobank. The solid line represents the significance threshold for all genetic variants (*p* < 5 x 10^-8^) and the dotted line the significant threshold for genetic variants in or nearby (±50 kb) candidate genes (*p* < 1 x 10^-6^).
