# Supplementary figures and images for: Association of common genetic variants with chronic axonal polyneuropathy in the general population: a genome-wide association study
Source: Front Neurol. 2024 Jul 3;15:1422824. doi: 10.3389/fneur.2024.1422824 (PMC11253699; doi:10.3389/fneur.2024.1422824)

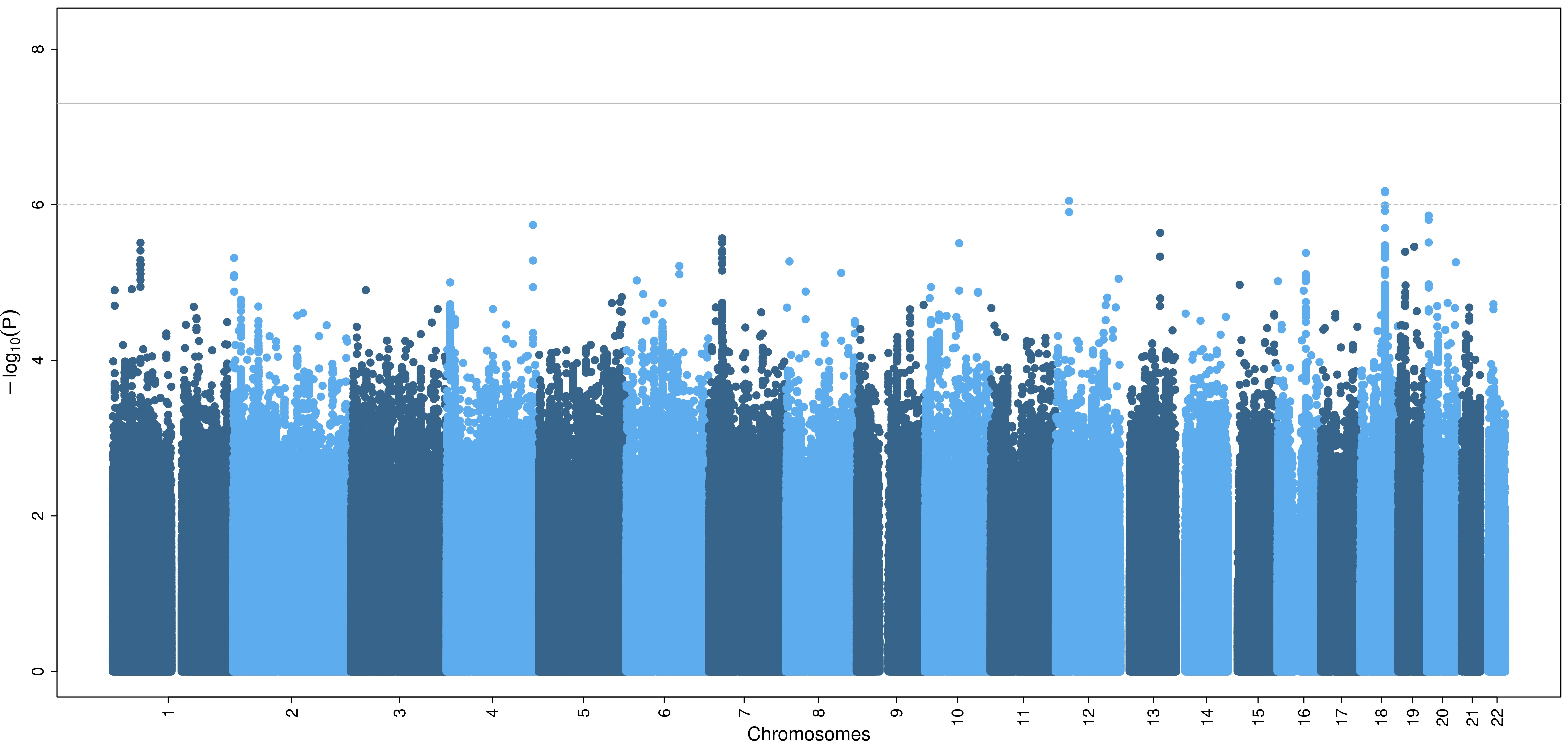

Supplement: SUPPLEMENTARY FIGURE 1 — Association of common genetic variants with chronic axonal polyneuropathy, including self-reported peripheral neuropathies in the UK Biobank. Manhattan plot showing the genome-wide association study for chronic axonal polyneuropathy, including the self-reported peripheral neuropathies in the UK Biobank. The solid line represents the significance threshold for all genetic variants (p < 5 x 10^-8^) and the dotted line the significant threshold for genetic variants in or nearby (±50 kb) candidate genes (p < 1 x 10^-6^). [file Image_1.JPEG]
