# Supplementary material for: Association of common genetic variants with chronic axonal polyneuropathy in the general population: a genome-wide association study
Source: Front Neurol. 2024 Jul 3;15:1422824. doi: 10.3389/fneur.2024.1422824 (PMC11253699; doi:10.3389/fneur.2024.1422824)
Supplement: Supplementary file 2 [file Table_1.DOCX]

**Supplementary table 1.** List of candidate genes, known to cause pure or predominant CMT.

| LMNA | BICD2 | SH3TC2 | MCM3AP | PRNP |
| --- | --- | --- | --- | --- |
| DNM2 | FBXO38 | NDRG1 | HARS | CCT5 |
| HSPB8 | SETX | PRX | NAGLU | FLVCR1 |
| CRYAB | FIG4 | HK1 | PMP2 | RTN2 |
| IGHMBP2 | SIGMAR1 | FGD4 | MTATP6 | SLC25A46 |
| PLEKHG5 | NEFH | GJB1 | DCAF8 | MTATP8 |
| HSPB1 | CHCHD10 | GJB3 | DGAT2 | MPV17 |
| HSPB3 | AR | AIFM1 | LAS1L | FAM134B |
| AARS | VRK1 | PRPS1 | C12orf65 | IKBKAP |
| GARS | SLC52A3 | PDK3 | DRP2 | WNK1 |
| BSCL2 | SLC52A2 | MFN2 | MYH14 | NGF |
| REEP1 | PMP22 | RAB7 | ATL3 | DNMT1 |
| SLC5A7 | MPZ | LRSAM1 | NTRK1 | SEPT9 |
| DCTN1 | LITAF | SPG11 | DST | GAN |
| ATP7A | EGR2 | MME | SCN11A | CTDP1 |
| DNAJB2 | NEFL | SCO2 | PRDM12 | TTR |
| TRPV4 | ARHGEF10 | ATP1A1 | SCN9A | TUBB3 |
| DYNC1H1 | FBLN5 | MORC2 | CLTCL1 | DHTKD1 |
| MTMR2 | YARS | MED25 | SPTLC1 | SURF1 |
| SBF2 | INF2 | HINT1 | SPTLC2 | TRIM2 |
| SBF1 | GNB4 | KARS | ATL1 | MARS |
| TFG | GDAP1 | COX6A1 | KIF1A | KIF5A |

**Supplementary figure 1. Association of common genetic variants with chronic axonal polyneuropathy, including self-reported peripheral neuropathies in the UK Biobank.**

Legend: Manhattan plot showing the genome-wide association study for chronic axonal polyneuropathy, including the self-reported peripheral neuropathies in the UK Biobank. The solid line represents the significance threshold for all genetic variants (p<5x10^-8^) and the dotted line the significant threshold for genetic variants in or nearby (± 50 kb) candidate genes (p<1x10^-6^).
